# γ-Aminobutyric acid as a biomarker of the lateralizing and monitoring drug effect in patients with magnetic resonance imaging-negative temporal lobe epilepsy

**DOI:** 10.3389/fnins.2023.1184440

**Published:** 2023-05-15

**Authors:** Shuohua Wu, Qianqi Wang, Huige Zhai, Yiwen Zhang, Dongyuan Xu, Gen Yan, Renhua Wu

**Affiliations:** ^1^Department of Radiology, The Second Affiliated Hospital of Xiamen Medical College, Xiamen, China; ^2^Department of Medical Imaging, The Second Affiliated Hospital, Medical College of Shantou University, Shantou, China; ^3^Department of Basic Medical Sciences, School of Medicine, Xiamen University, Xiamen, China; ^4^Center of Morphological Experiment, Medical College of Yanbian University, Jilin, China; ^5^Department of Neurology, The Second Affiliated Hospital of Xiamen Medical College, Xiamen, China

**Keywords:** GABA, lateralization, seizure, temporal lobe epilepsy, ^1^H-MRS

## Abstract

**Introduction:**

Despite verifying proton magnetic resonance spectroscopy (^1^H-MRS) for focal localization in magnetic resonance imaging (MRI)-negative temporal lobe epilepsy (TLE), it is necessary to illustrate metabolic changes and screen for effective biomarkers for monitoring therapeutic effect. We used ^1^H-MRS to investigate the role of metabolic levels in MRI-negative TLE.

**Materials and methods:**

Thirty-seven patients (*n* = 37, 14 women) and 20 healthy controls (*n* = 20, 11 women) were investigated by ^1^H-MRS. We compared the metabolite level changes in the epileptic and contralateral sides on the mesial temporal and dorsolateral prefrontal cortices and analyzed their association with clinical symptoms.

**Results:**

γ-Aminobutyric acid (GABA) levels were significantly lower on the epileptic side (2.292 ± 0.890) than in the contralateral side (2.662 ± 0.742, *p* = 0.029*) in patients on the mesial temporal lobe. N-acetylaspartate (NAA) levels were significantly lower on the epileptic side (7.284 ± 1.314) than on the contralateral side (7.655 ± 1.549, *p* = 0.034*). NAA + N-acetylaspartylglutamate levels were significantly lower on the epileptic side (7.668 ± 1.406) than on the contralateral side (8.086 ± 1.675, *p* = 0.032*). Glutamate levels were significantly lower on the epileptic side (7.773 ± 1.428) than on the contralateral side (8.245 ± 1.616, *p* = 0.040*). Moreover, a significant negative correlation was found between GABA levels in the epileptic mesial temporal lobe and tonic–clonic seizure frequency (r = −0.338, *p* = 0.046*).

**Conclusion:**

γ-Aminobutyric acid (GABA) is a potential biomarker for lateralization and monitoring seizure frequency in MRI-negative TLE.

## 1. Introduction

Temporal lobe epilepsy (TLE) is a common epileptic syndrome. Up to 30% of patients with TLE are magnetic resonance imaging (MRI)-negative. Moreover, 70% of patients with TLE have a high risk of developing drug resistance (Muhlhofer et al., 2017). The effectiveness of pharmacological and surgical treatments depends on epilepsy type, underlying pathology, and accurate location of the epileptogenic brain region by various clinical, neuroimaging, and neurophysiological investigations (Ryvlin et al., 2014). To this end, electroencephalography (EEG) and intracranial EEG (IEEG) are essential tools for the location and lateralization of MRI-negative TLE epileptic foci, especially IEEG. However, IEEG is invasive, and EEG and IEEG fail to dynamically monitor therapeutic effects (Jayakar et al., 2016; Hamelin et al., 2021).

The initial brain damage of TLE results in hippocampal sclerosis followed by collateral axonal sprouting and synaptic circuitry reorganization, eventually affecting the balance between inhibition and excitation in limbic circuits until initial seizure onset (Malmgren and Thom, 2012; Thijs et al., 2019). For TLE, the networks involve neuronal circuits in one hemisphere, commonly limbic or neocortical (Bilevicius et al., 2010; Fisher et al., 2017). Epileptogenic results from an imbalance between the excitatory and inhibitory activities within a neuronal network (Davis et al., 2015; Sarlo and Holton, 2021). The underlying pathophysiological basis of changes in neuronal excitation and inhibition remains unclear.

γ-Aminobutyric acid (GABA) is the principal inhibitory neurotransmitter in the cerebral cortex and maintains an inhibitory tone that counterbalances neuronal excitation (Wong et al., 2003). A disturbance of this balance may lead to seizures. Recently, proton magnetic resonance spectroscopy (^1^H-MRS) is the most essential and noninvasive method used to detect specific brain metabolites (Di Costanzo et al., 2003). The Mescher–Garwood point resolved spectroscopy sequence (MEGA-PRESS) can detect GABA from other metabolites (Mullins et al., 2014). A decrease in N-acetylaspartate (NAA) or its ratio to other metabolites is feasible for lateralizing the side of MRI-negative TLE (Connelly et al., 1998; Woermann et al., 1999; Hammen et al., 2007). However, there is limited evidence for changes in metabolite levels in epileptic regions associated with clinical symptoms and treatment effects.

In our study, we aimed to use ^1^H-MRS to analyze metabolite level changes in the mesial temporal cortex (MTLC) and dorsolateral prefrontal cortex (DLPFC) in patients with MRI-negative TLE and to explore the association between metabolite levels and ages, ages at epilepsy, duration of epilepsy, tonic–clonic seizure (TCS) frequency, and interval days since the most recent seizure. We aimed to verify the correlation between metabolite levels and epileptic foci suggested by video-EEG and to continually explore the association between the aforementioned factors and changes in metabolites in the epileptic foci based on previous studies.

## 2. Materials and methods

### 2.1. Participants

All examinations were performed with the written consent of each participant, and this study was approved by the Ethics Department. All procedures were conducted according to the principles of the Declaration of Helsinki.

In our article, we define “MRI-negative TLE” to represent a disorder where recurrent unprovoked seizures occur with or without secondary generalization, originating from the temporal lobe based on electroclinical findings, in the absence of an epileptogenic lesion on visual inspection of the MRI. Seizures may originate from the mesial or lateral temporal regions (or simultaneously from both), sometimes with variable extension of the epileptogenic zone (EZ) to include the neighboring lobes (“temporal-plus” epilepsy; Muhlhofer et al., 2017).

We prospectively assessed 37 patients with TLE who attended the Department of Neurology at the hospital between April 2019 and January 2022. The classification of TLE followed the International League Against Epilepsy criteria (Scheffer et al., 2017). All patients were diagnosed with focal TLE by two experienced neurologists, based on the available data [history, seizure description on video and by expert witnesses, and prolonged interictal video electroencephalogram (EEG)] and thorough neuropsychological evaluations. The inclusion criteria were as follows: (1) non-lesional TLE based on MRI scans; (2) unprovoked seizures occurring with or without secondary generalization, originating from the temporal lobe based on video-EEG; and (3) seizures possibly originating from the mesial or lateral temporal region (or simultaneously from both), sometimes with the variable extension of the epileptogenic zone to include the neighboring lobes (“temporal-plus” epilepsy). The exclusion criteria were as follows: (1) other non-epileptic seizure diseases, such as pseudoseizure and febrile convulsion; (2) hippocampal sclerosis, traumatic brain injury, and dual pathology; and (3) hysteria, Parkinson’s disease, depression, and other mental or neurological disorders.

We examined 37 patients with focal TLE who were MRI-negative [14 women; median age, 28.5 (range, 19–55) years] and 20 healthy controls [HCs; 11 women; median age, 28 (range, 21–57) years] using a 3.0-T GE scanner and standard 24-channel head surface coil (Discovery MR750, GE Healthcare, Milwaukee, WI, United States). The inclusion criteria of HCs were as follows: (1) The general health condition is good, without organic or neuropsychiatric disease history or family history; (2) The participants’ immediate family members have no history of epilepsy; (3) No abnormal lesions or anatomical variations were found in the MRI structural images. There was no significant difference in sex and age distribution between patients and HCs.

### 2.2. Clinical evaluation

**Figure 1:**
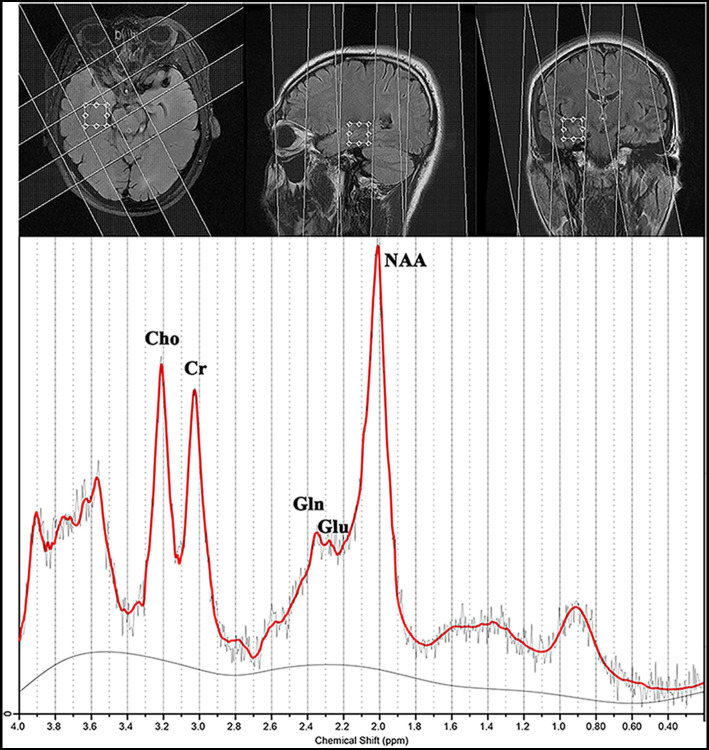
Magnetic resonance spectroscopy findings for a patient with magnetic resonance imaging-negative temporal lobe epilepsy.

Medical and neurological history and physical examination were performed for all participants. The ages, seizure-onset age, duration of epilepsy, TCS frequency, anti-epileptic drug (AED) treatment, interval days since the most recent seizure, and interictal surface video-EEG were recorded (Supplementary Table S1). Figure 1 shows a diagram of the study protocol.

### 2.3. Video-EEG methods

We recorded the scalp video-EEG using an international 10 to 20 system with 21 electrodes. The interictal EEG was recorded during in-patient video-EEG monitoring, sleep and wake cycles, and routine activation procedures (i.e., hyperventilation and photic stimulation). We reviewed the entire interictal EEG recording for evidence of generalized or partial epileptiform discharges. The localization of interictal EEG was based on the region of onset of rhythmic seizure activity.

### 2.4. MRI study

The initial MRI protocol included 3-mm-thick slices in all three directions [axial T2-weighted fast spin-echo (FSE), coronal T2, and sagittal T2 fluid-attenuated inversion recovery (FLAIR)]. We acquired axial T2-weighted FSE images [echo time/repetition time (TE/TR), 102/3,500 ms; matrix, 256 × 256; number of excitations (NEX), 2; frequency field of view (FOV), 240 mm; and phase FOV, 7.5 mm]. Moreover, we acquired coronal T2 FLAIR images (TE/TR, 145/9,000 ms; matrix, 320 × 256; NEX, 1; frequency FOV, 240 mm; and phase FOV, 10 mm) and sagittal T2 FLAIR images (TE/TR, 145/5,000 ms; matrix, 320 × 256; NEX, 1; frequency FOV, 240 mm; and phase FOV, 10 mm). High-resolution MR imaging was performed using a 3-D BRAVO sequence (BRAin Volume; TE/TR, 2.4/7.0 ms; matrix, 256 × 256; NEX, 1; frequency FOV, 240 mm; phase FOV, 10 mm; flip angle, 12°; and slice thickness, 1 mm). Two experienced neuroradiologists blinded to the clinical, EEG, and other imaging data reviewed all MRI studies and independently analyzed the diagnostic MR scans [Advantage Workstation 4.6 (GE Healthcare)]. They evaluated and excluded the following MRI criteria of focal cortical dysplasia: increased cortical thickness, blurring of the gray matter–white matter junction, abnormal gyration, and FLAIR/FSE/3-D Bravo white and gray matter signal changes.

### 2.5. Routine MRS

Routine ^1^H-MRS was performed within 1–2 weeks before video-EEG monitoring. A single voxel was prescribed from the axial T2FSE images in the bilateral mesial temporal lobes of each participant with 20 mm in the anteroposterior and left–right directions, and we selected a thickness of 20 mm to avoid spectroscopic artifacts caused by the proximity to regions of high magnetic susceptibility difference (Figure 1).

Proton magnetic resonance spectroscopy was obtained in the transversal plane using a volume preselected PRESS-CSI hybrid sequence as follows: FOV, 240 × 240 mm; 16 × 16 steps; TR/TE, 3,000/35 ms; one acquisition; slice thickness, 20 mm; and nominal voxel volume, 20 × 20 × 20 mm^3^. We collected two spectra in the bilateral mesial temporal lobes for 14 min.

### 2.6. Mega-press

**Figure 2:**
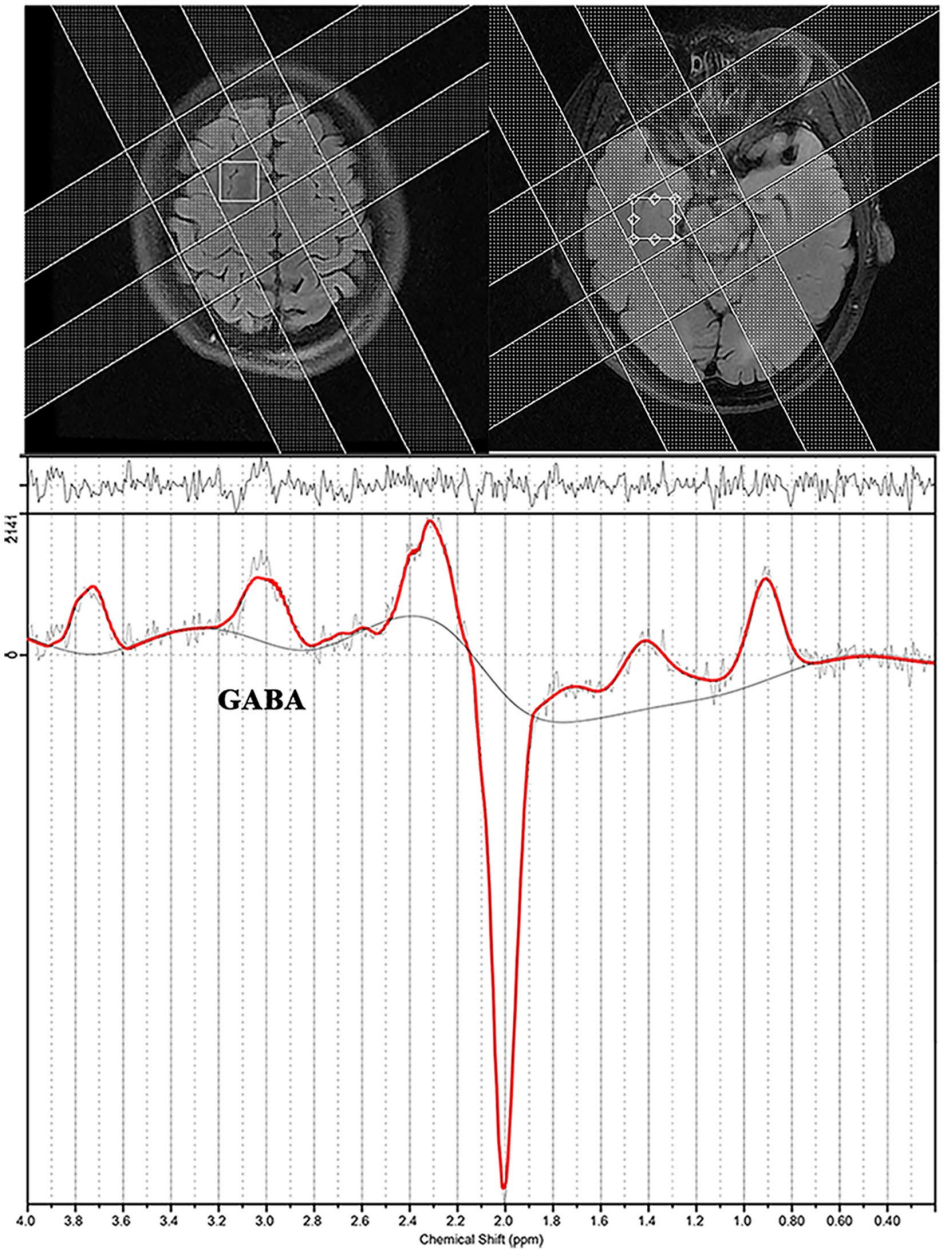
Screenshot from an LCModel depicting an example of the spectra acquired from a patient with epilepsy.

Single-voxel edited ^1^H MR spectra were acquired from a 20 × 30 × 30 mm^3^ voxel of interest positioned in the bilateral DLPFC and MTLC using MEGA-PRESS (Figure 2). We selected the DLPFC and MTLC as the target voxels in the abnormal discharge areas, indicated by the interictal video scalp EEG. Figure 2 depicts the screenshots of the DLPFC and MTLC voxels. In total,320 spectral averages were acquired for each spectrum, with a TR and TE of 1,800 ms and 68 ms, respectively.

### 2.7. MRS data analysis

The spectra were coiled with weighting factors derived from the first point of the free induction decay signal from non-water-suppressed acquisitions of each coil. We derived water-scaled metabolite concentrations from the edited spectra using a linear combination of the model spectra (LCModel) with a simulated basis set, including the basis spectra for glutamine (Gln), glutamate (Glu), NAA, and N-acetylaspartylglutamate (NAAG).

For GABA quantification, we used the control parameter sptype = “MEGA-PRESS-2” for the LCModel analysis (Section 3.1 of the LCModel manual). Factors affecting the signal intensity in MEGA-PRESS, such as J-coupling effects and T2 losses, implied that the absolute values may have an additional (unknown) scaling factor and should be considered institutional units rather than absolute values.

The uncertainties estimated by Cramer–Rao Lower Bounds served as the primary guidelines for arbitrating the spectra of absolute metabolite concentrations. Only metabolite spectra with an LCModel estimated an uncertainty <15% standard deviation (SD) and spectra with a signal-to-noise ratio > 3 were included in this study. We used a constrained regularization method to estimate the line shape and baseline accounting for the residual water signal. This study did not have spectra with a full width at half maximum >15 Hz.

### 2.8. Statistical analyses

All data were analyzed using the SPSS Statistics Package (version 25.0; IBM Corporation, New York, NY, United States). We determined the normality of the distribution of metabolite concentrations in patients and control participants using the Kolmogorov–Smirnov test. The normal ranges were established and are expressed as control mean ± SD. Subsequently, we used a significance level of 0.05. We performed the two-sample paired t-test to compare the epileptic and non-epileptic sides of patients and both hemispheres of the normal brain. Moreover, linear correlation analyses were performed to investigate the association among ages, ages at epilepsy, duration of epilepsy, TCS frequency, interval days since the most recent seizure, and metabolite concentrations (Pearson’s correlation); to compare regular treatment patients with those with irregular treatment; and to compare seizure-free patients with those with ongoing seizures (two-tailed unpaired t-test).

## 3. Results

### 3.1. Comparison of epilepsy and contralateral sides

**Figure 3:**
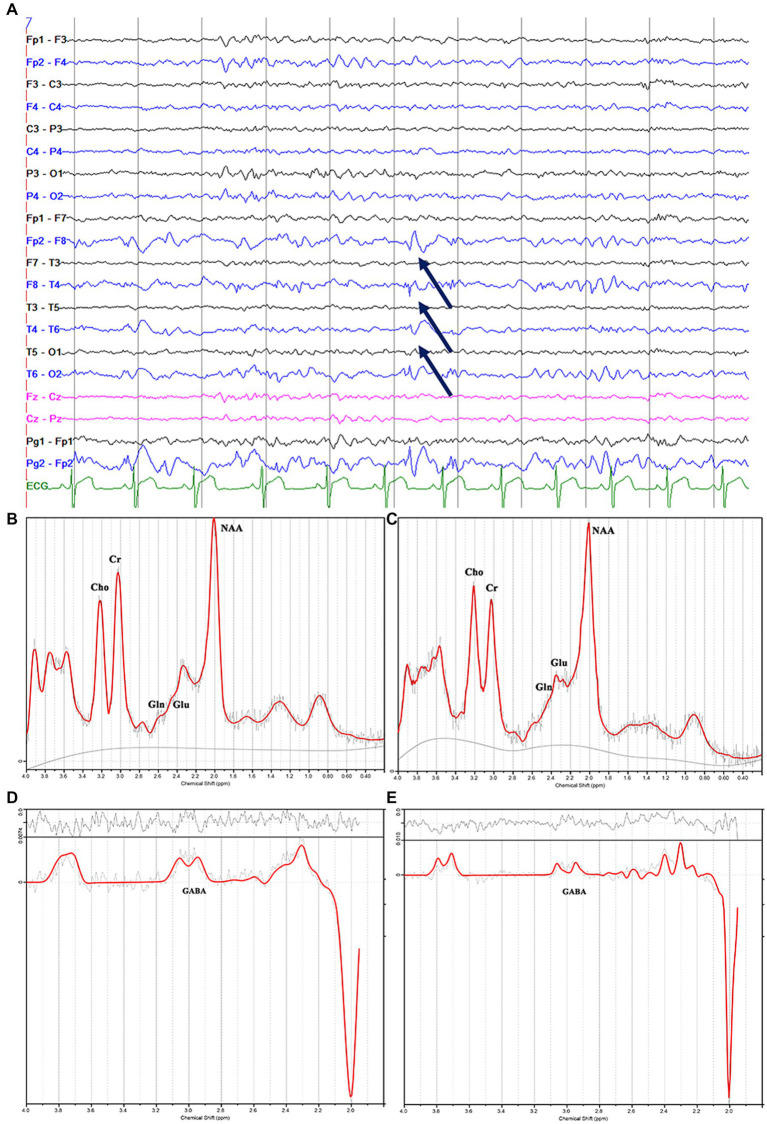
Electroencephalography pattern of interictal activity (interictal epileptiform discharges; arrows) localized in the right hemisphere **(A)**; **(C)** and **(E)** show the decreased N-acetylaspartate, glutamate, and γ-aminobutyric acid peaks in the right mesial temporal lobe, and **(B)** and **(D)** show the contralateral hemisphere in the same patients with magnetic resonance imaging-negative temporal lobe epilepsy.

**Figure 4:**
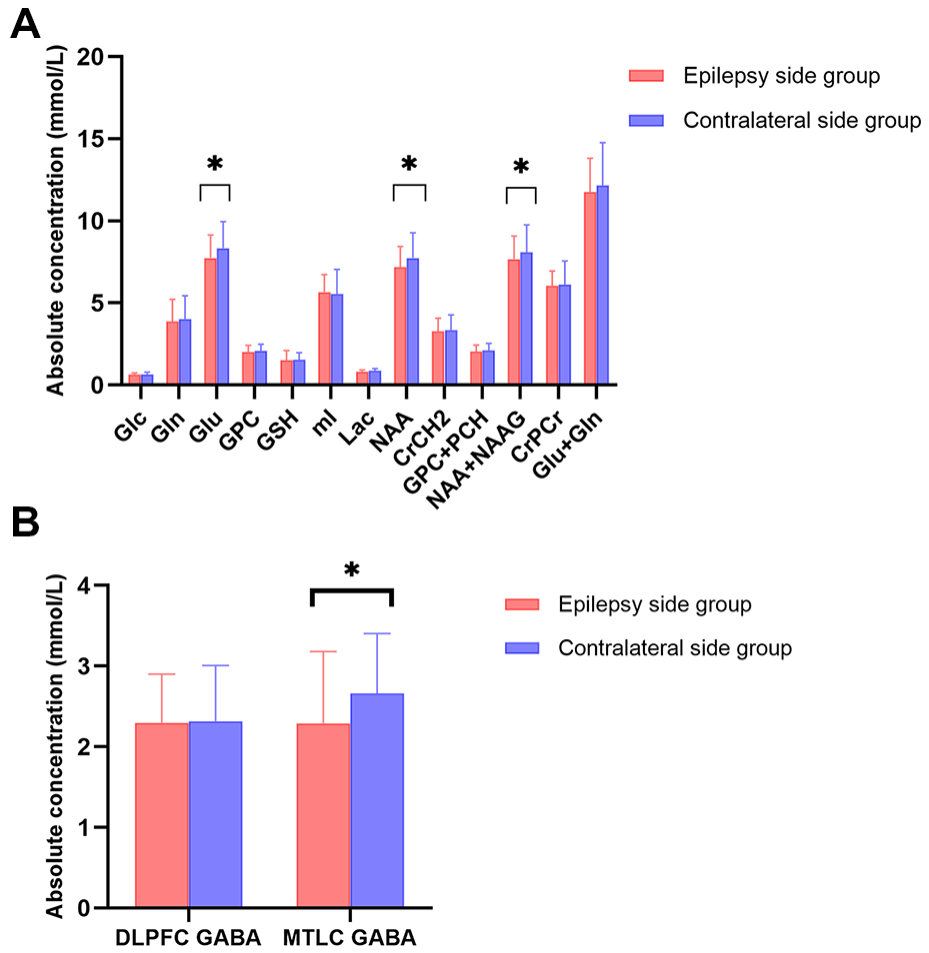
**(A)** Bar plots displaying the values for different metabolites in the mesial temporal cortex (MTLC) on the epilepsy side (red) and contralateral side (blue); **(B)** Bar plots displaying metabolite values for γ-aminobutyric acid concentration in the dorsolateral prefrontal cortex and MTLC concentrations on the epilepsy side (red) and contralateral side (blue) Error bars denote standard deviations, and significant differences are indicated by asterisks (*p < 0.05, **p < 0.01, and ***p < 0.001).

In the MRI-negative epilepsy group, 37 participants (*n* = 37, 14 women) met all inclusion criteria. Paired t-tests were conducted to examine the metabolite symmetry of the epilepsy side (video-EEG localization) and contralateral side. In the mesial temporal lobe, GABA, NAA, NAA + NAAG, and Glu concentrations were lower in the epilepsy side voxel than in the contralateral side voxel (*p* < 0.05). Specifically, GABA levels were significantly lower on the epileptic side (2.292 ± 0.890) than on the contralateral side (2.662 ± 0.742, *p* = 0.029*). NAA levels of the epileptic side were significantly lower (7.284 ± 1.314) than those of the contralateral side (7.655 ± 1.549, *p* = 0.034*). NAA + NAAG levels were significantly lower on the epileptic side (7.668 ± 1.406) than on the contralateral side (8.086 ± 1.675, *p* = 0.032*). Glu levels were significantly lower on the epileptic side (7.468 ± 1.294) than on the contralateral side (8.682 ± 1.435, *p* = 0.040*). GABA, NAA, NAA + NAAG, and Glu level changes were related to the lateralization of the video-EEG (Table 1; Figure 3). In the prefrontal lobe cortex, GABA concentrations in the epilepsy side voxel were lower than those in the contralateral side voxel (epilepsy side 2.299 ± 0.602 vs. contralateral side, 2.317 ± 0.691); however, the difference was statistically insignificant (*p* = 0.919 and *p* > 0.05, respectively; Figure 4).

**Table 1:** The absolute concentration of metabolites in patients and healthy controls.

	Patient group			Healthy control group		
	Epilepsy side Mean ± SD	Contralateral side Mean ± SD	*p*-value	Right side Mean ± SD	Left side Mean ± SD	*p*-value
Gender (women)	37 (14)			20 (9)		0.834
Median age (range)	28.5 (19–55)			26 (21–58)		0.728
DLPFC GABA	2.299 ± 0.602	2.317 ± 0.691	0.919	2.550 ± 0.552	2.758 ± 0.545	0.316
MTLC GABA	2.292 ± 0.890	2.662 ± 0.742	**0.029** *	2.894 ± 0.482	2.650 ± 0.495	0.483
MTLC Glc	0.636 ± 0.066	0.665 ± 0.078	0.871	0.345 ± 0.069	0.348 ± 0.046	0.982
MTLC Gln	3.867 ± 1.359	4.030 ± 1.414	0.668	3.395 ± 1.256	3.223 ± 1.222	0.618
MTLC Glu	7.773 ± 1.428	8.245 ± 1.616	**0.040** *	7.685 ± 0.958	7.962 ± 1.041	0.363
MTLC GPC	2.027 ± 0.402	2.092 ± 0.401	0.316	2.001 ± 0.602	1.944 ± 0.308	0.634
MTLC GSH	1.517 ± 0.590	1.541 ± 0.439	0.860	1.435 ± 0.537	1.375 ± 0.488	0.670
MTLC mI/Ins	5.666 ± 1.058	5.538 ± 1.511	0.665	5.147 ± 1.058	5.142 ± 0.999	0.983
MTLC Lac	0.826 ± 0.057	0.869 ± 0.072	0.807	0.885 ± 0.087	0.588 ± 0.035	0.120
MTLC NAA	7.284 ± 1.314	7.655 ± 1.549	**0.034** *	7.949 ± 1.138	8.112 ± 0.919	0.500
MTLC CrCH2	3.281 ± 0.800	3.336 ± 0.938	0.730	3.486 ± 0.904	3.161 ± 0.624	0.175
MTLC GPC + PCH	2.041 ± 0.403	2.112 ± 0.428	0.370	1.898 ± 0.427	1.943 ± 0.308	0.516
MTLC NAANAAG	7.668 ± 1.406	8.086 ± 1.675	**0.032** *	8.021 ± 0.889	8.293 ± 0.883	0.244
MTLC Cr + PCr	6.035 ± 0.912	6.125 ± 1.430	0.736	5.486 ± 0.883	5.779 ± 0.593	0.179
MTLC Glu + Gln	11.80 ± 2.030	12.27 ± 2.614	0.414	11.32 ± 1.892	11.29 ± 1.816	0.870

* MTLC, mesial temporal lobe cortex; DLPFC, dorsolateral prefrontal cortex; GABA, γ-Aminobutyric acid; Gln, glutamine; Glu, glutamate; GPC, glycerylphosphorylcholine; mI/Ins, myo-inositol; PCH, phosphorylcholine; –CrCH2, Cr methylene group; GSH, glutathione; Cr, creatine; PCr, Phosphocreatine; Lac, lactic acid; NAA, N-acetylaspartate; and NAAG, N-acetylaspartylglutamate.

Significant differences are indicated by asterisks (*p < 0.05, ** *p* < 0.01, and *** *p* < 0.001). The bold values refer to statistically significant differences.

In the HC group (*n* = 20, 11 women), we performed two-sample paired t-tests to examine the right–left-side metabolite symmetry, and the differences were not statistically insignificant (*p* > 0.05). Metabolic concentrations in the HC group were roughly symmetric (Table 1).

### 3.2. Association with clinical indicators

We used linear regression analysis to explore the effect of multiple clinical measures, including gender factors, on specific metabolites. Firstly, Pearson correlation coefficients (PCCs) method to analyze the correlation between male and female gender, age of the patient, age at initial seizure onset, duration of disease, TCS frequency, the interval days since the last TCS.” A strong correlation was found between male and female gender. Secondly, we incorporated these factors into the linear regression equation for consideration. We found that for the GABA level in epileptic MTLC, except TCS frequency (*p* = 0.071) closed to *p* < 0.05, all other factors including gender were *p* > 0.05 (Supplementary Table S2).

### 3.3. Association with tonic–clonic seizures

**Figure 5:**
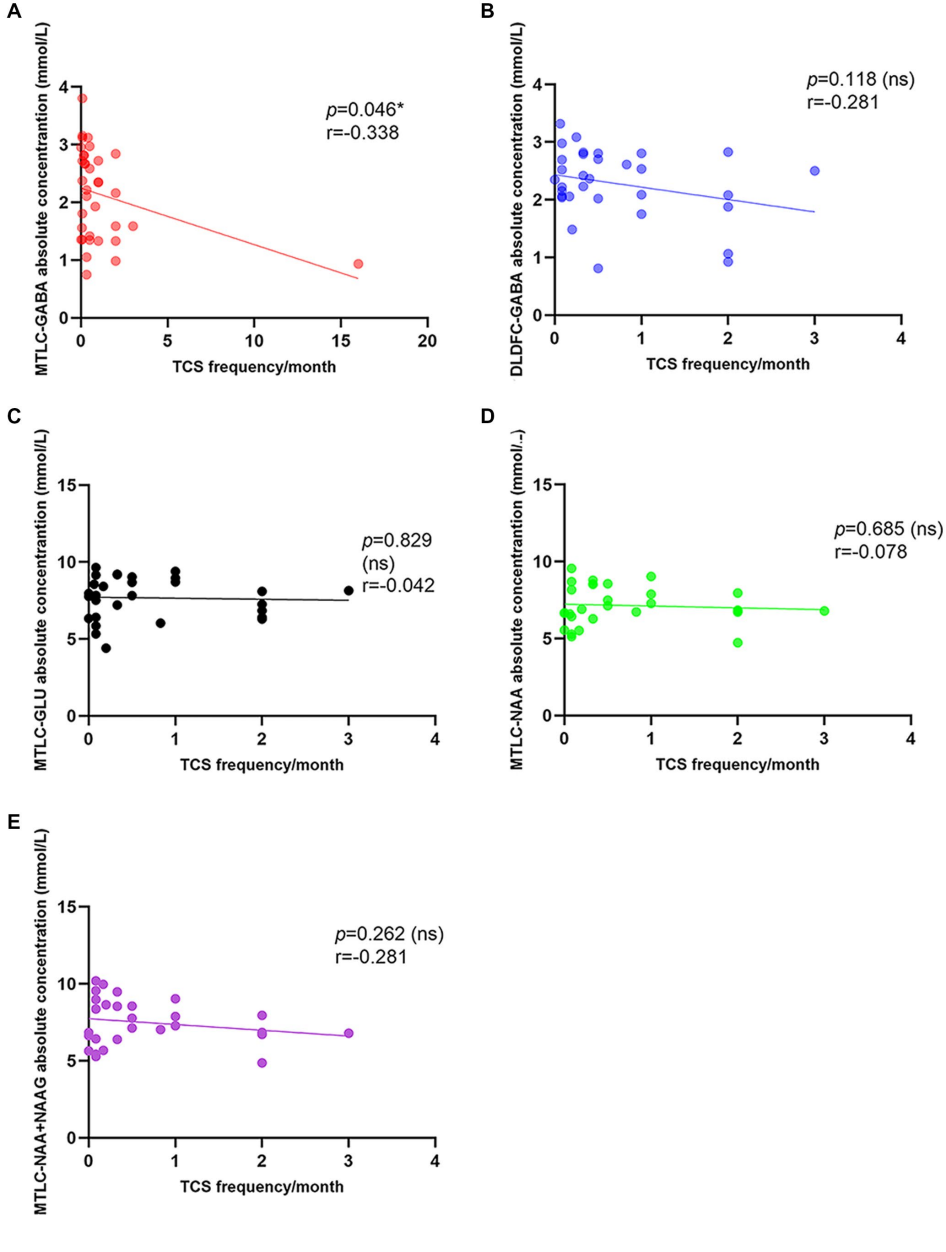
Correlation between tonic–clonic seizure frequency and metabolite levels in the epileptic hemisphere. **(A)** Correlation between tonic-clonic seizure frequency and GABA levels in the epileptic MTLC; **(B)** Correlation between tonic-clonic seizure frequency and GABA levels in the epileptic DLPFC; **(C)** Correlation between tonic-clonic seizure frequency and Glu levels in the epileptic MTLC; **(D)** Correlation between tonic-clonic seizure frequency and NAA levels in the epileptic MTLC; **(E)** Correlation between tonic-clonic seizure frequency and NAA+NAAG levels in the epileptic MTLC.

A significant low correlation was found between TCS frequency and GABA levels in epileptic MTLC (r = −0.338; *p* = 0.046*; Figure 5). Moreover, there were no significant associations for GABA levels in epileptic DLPFC and other metabolites levels in epileptic MTLC with TCS frequency.

### 3.4. Association with gender, age of patients, age of initial seizure, duration of disease, the interval days since the last TCS

There were no significant associations for any metabolite with ages, age at epilepsy onset, duration of epilepsy, or interval days since the most recent seizure.

### 3.5. Association with AED regular therapy

**Figure 6:**
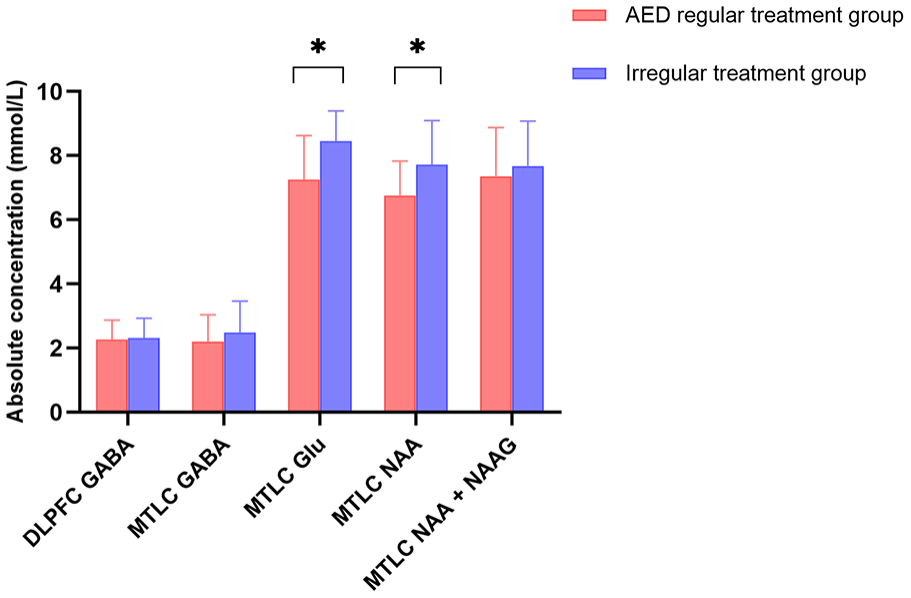
Absolute concentrations of metabolites in the regular treatment (red) and irregular treatment (blue) groups. Error bars denote standard deviations, and significant differences are indicated by asterisks (*p < 0.05, **p < 0.01, and ***p < 0.001).

When the AED regular treatment group was compared with the irregular treatment group for metabolites that could be used to lateralize, the irregular treatment group in epileptic MTLC had significantly higher Glu levels (*p* = 0.020*) than the AED regular treatment group. Moreover, the irregular treatment group had higher NAA levels than the AED regular treatment group (*p* = 0.053; Table 2; Figure 6). GABA levels in epileptic MTLC (*p* = 0.376) and DLPFC (*p* = 0.815) were lower in the regular treatment group than in the irregular treatment group, but the difference was not statistically significant (*p* > 0.05).

**Table 2:** The absolute concentration of metabolites in AED regular treatment and irregular treatment groups.

	Regular treatment	Irregular treatment	*P*-value
Number	26	11	
Age(years)	32.20 ± 12.56	28.67 ± 10.33	0.477
Metabolites concentrations (mean ± SD)			
DLPFC GABA	2.262 ± 0.606	2.316 ± 0.615	0.815
MTLC GABA	2.200 ± 0.695	2.490 ± 0.972	0.376
MTLC Glu	7.256 ± 1.371	8.458 ± 0.940	**0.020** *
MTLC NAA	6.795 ± 1.064	7.717 ± 1.379	0.053*
MTLC NAA + NAAG	7.364 ± 1.514	7.679 ± 1.401	0.590

* MTLC, mesial temporal lobe cortex; DLPFC, dorsolateral prefrontal cortex; GABA, γ-aminobutyric acid; Glu, glutamate; NAA, N-acetylaspartate; and NAAG, N-acetylaspartylglutamate.

Significant differences are indicated by asterisks (*p < 0.05, **p < 0.01, and ***p < 0.001). The bold values refer to statistically significant differences.

## 4. Discussion

To the best of our knowledge, this is the first study to investigate *in vivo* MTLC and DLPFC GABA levels in MRI-negative TLE using MEGA-PRESS with macromolecule suppression simultaneously. This is also the largest study of GABA levels in MRI-negative TLE measured with MRS to date.

Our primary findings suggest that reductions in GABA, NAA, NAA + NAAG, and Glu concentrations in MTLC can be used to lateralize patients with MRI-negative epilepsy, consistent with EEG lateralization. Moreover, a significant low correlation was found between GABA levels in epileptic MTLC and TCS frequency.

Abnormalities in the GABAergic system have been implicated in the pathogenesis of epilepsy (Treiman, 2001). Strikingly abnormal GABA concentrations have been documented in some patients with intractable epilepsy (Chang et al., 2003). Concomitantly, a recent study reported similar findings, and the GABA/Cr ratio decreased bilaterally in the MTLC in patients with TLE at an early stage (He et al., 2021). In previous *in vivo* measurements, patients with poorer seizure control tended to display lower GABA levels, similar to our study (Chang et al., 2003). Few studies have investigated GABA concentrations, and one study reported on increased GABA in a different brain region (Sarlo and Holton, 2021). Patients with idiopathic generalized epilepsy display higher frontal Gln and GABA levels compared with controls (Chowdhury et al., 2015). In an *in vivo* animal study, researchers observed a significant increase in GABA concentration on the epileptic side of the MTLC (Hamelin et al., 2021). However, these studies reported opposite results, either.

Positron emission tomography scans utilizing 2-[18F] fluoro-2-deoxy-D-glucose positron emission tomography (FDG-PET) and other ligands can help identify epilepsy-related metabolic disturbances when MRI is negative. The underlying pathophysiologic basis for the reduction in glucose uptake (relative focal or regional hypometabolism) seen in the interictal state is still unresolved. Other PET tracers besides FDG may be useful in the evaluation of MRI-negative TLE. These include [11C] flumazenil (FMZ), which shows decreased GABAA receptor binding in the presumed epileptic focus, demonstrating a more restricted region of hypometabolism compared with FDG-PET. The decreased GABA levels in epigenetic region is consistent to PET GABA-specific imaging ([11C] FMZ). Impaired GABA function can lead to the imbalance between excitation and inhibition, thus causing seizures (Sears and Hewett, 2021). Chronic loss of GABAergic function and abnormalities in GABA receptors in the epileptogenic human cortex is supported by biochemical analysis of tissue resected for the resolution of focal seizures (Shetty and Upadhya, 2016).

γ-Aminobutyric acid, Gln, and Glu are interconvertible and dynamically balanced (Zhou and Danbolt, 2014). GABA is synthesized in neurons from Glu, with Gln production being an intermediate step via glutamic acid decarboxylase and the cofactor pyridoxal phosphate (Petroff, 2002). Glial Gln can be transported back into neurons to synthesize GABA (Albrecht and Zielińska, 2017; Boison and Steinhäuser, 2018). Studies of seizure-onset patterns *in vivo* and *in vitro* have demonstrated that increased activity in excitatory neurons and inhibitory interneurons initiate seizures (Schevon et al., 2012). This neuronal activity pattern during low-voltage fast activity was confirmed by unit recordings in patients with epilepsy. Enhanced inhibitory activity before a focal seizure may indicate an attempt at seizure prevention (Schevon et al., 2012; Elahian et al., 2018). Therefore, we observed a simultaneous increase and decrease in GABA and Glu/Gln levels in epileptogenic region in our study and other studies (Chowdhury et al., 2015).

In our study, both GABA level and Glu level were Simultaneous decreased in the epileptogenic zone, which is very worth exploring. According to the traditional theory, the equilibrium between excitation and inhibition is broken, and the inhibitory neurotransmitter decreases, accompanied by the increase of excitatory neurotransmitter (Petroff, 2002). For the unique phenomenon observed in this study, the possible explanations were as following: (1) Seizures are self-terminating events that use homeostatic feedback mechanisms induced by the ictal discharge to terminate (Lado and Moshé, 2008). During the later stages of focal seizures, the firing of principal cells and GABAergic neurons is enhanced and synchronized in spike bursts. The inhibition state after the burst prevents re-excitation to prevent seizures (Farrell et al., 2017). The brain also has its own protective mechanism for seizure states, and studies have demonstrated that impairment of this protective mechanism may be one of the causes of drug-refractory epilepsy. (2) In the presence of Glu-Gln cycle, Glu is decreased, but Gln is increased in the epileptogenic zone. We speculate that part of glutamate is converted into Gln in this process. (3) It is not clear whether the GABA and Glu measured by MRS are extracellular or intracellular, or both. In theory, it is extracellular neurotransmitters that act on the balance of excitation and inhibition (Bell et al., 2021). Therefore, a decrease in GABA accompanied by a decrease in Glu may only be a decrease in extracellular neurotransmitters. Rather than an “overall” reduction.

Glu levels on the epileptic side were significantly lower than those on the contralateral side in our study, Gln levels on epileptic MTLC was higher than the contralateral side, although the difference was not statistically significant. Glu represents increased epileptic activity, and Gln is an astroglia-derived precursor of the neurotransmitter Glu. The Glu-Gln cycle hypothesis can explain the observed effects. In certain types of epilepsies, such as focal structural epilepsies, the impaired functioning of the Glu-Gln cycle supposedly results in neurotoxic increases in extracellular Glu (Eid et al., 2004; Devinsky et al., 2013), supported by a microanalytical study (Van Der Zeyden et al., 2008). Normal functioning of the Glu-Gln cycling can result in the conversion of increased Glu to Gln to maintain circulatory balance, even if the transiently elevated Glu in the epileptogenic focus, during the course of our measurements, has been converted to Gln (Cavus et al., 2005). In our study, an overall increase in the Glu + Gln levels of epileptogenic MTLC. When Glu level decreased, increased Gln maintains this dynamic equilibrium.

Moreover, AED treatment is also important, affecting metabolite levels. Twenty-six patients had AED regular treatment. Meanwhile, 11 patients had irregular treatment, whose GABA and Glu levels were higher than those with AED regular treatment, with their increasing Glu level statistically significant. Glutamate synthesis was interfered by AEDs (Sitges et al., 2007; Doelken et al., 2010), and the Glu level of the AED mediation group decreased significantly; a corresponding decrease in GABA level is consistent with a previous conclusion.

Interestingly, our study found a significant low correlation between GABA levels in the epileptic mesial temporal lobe and generalized TCS frequency (r = −0.338, *p* = 0.046*). Seizure worsening was defined as an increase in total average monthly TCS frequency, and the reduction of TCS frequency is an important indicator to evaluate therapeutic effects (Sarkis et al., 2012).

The results of all studies, including ours, demonstrate that the reduction of NAA + NAAG level is the most reliable metabolite alteration in lateralizing the epileptic hemisphere in patients with MRI-negative TLE (Simister et al., 2003; Shih et al., 2004; Hammen et al., 2006, 2007; Simister et al., 2009). Decreased NAA + NAAG levels in patients may have resulted from oxidative stress in neurons exhibiting increased electrical irritability (Pottoo et al., 2019).

In the present study, GABA concentrations of the epileptic side in the MTLC and DLPFC were significantly lower than those in HCs. However, only GABA in the mesial temporal region indicated the lateralization of MRI-negative TLE. According to previous studies, several patients with seizure origin and MRI abnormalities have unilateral seizures (Connelly et al., 1998; Suhy et al., 2002; Zuberi and Brunklaus, 2018). This finding is consistent with postmortem studies that reported on up to 50% of bilateral abnormalities and pathology findings, thus suggesting that the abnormalities often extend beyond the mesial temporal lobe in patients with TLE (Falconer, 1974). This further confirms that abnormal GABA concentrations represent a greater diffuse epileptogenic network in patients with MRI-negative TLE.

The reasoning behind analyzing the dorsolateral prefrontal cortex was as following. Firstly, in 16 patients, the epileptic seizure waves originated from the MTLC and projected to the DLPFC, which is also the brain region affected by epileptic waves that neurologists are concerned about. Secondly, In MRI-negative TLE patients, it is thought that there is a much larger diffuse epileptogenic network, which can spread from the seizure focus (mainly from the medial temporal lobe) to distant regions such as the DLPFC (Tan et al., 2018). Compared with the controls, TLE patients showed significantly decreased NAA and Ins, and the reductions were greater in the left DLPFC. This study suggests that TLE can produce metabolic changes to DLPFC that is remote from the seizure focus (Qin et al., 2020). Besides, in a Longitudinal Resting-State fMRI Study by Qin et al. (2020), cross-sectional analysis demonstrated that TLE patients displayed significantly greater positive correlation than NCs between the right dorsolateral prefrontal cortex (DLPFC) and the right inferior parietal lobule (IPL) and right superior frontal gyrus (SFG). Furthermore, among TLE patients, the longitudinal study revealed a decrease in correlation between the right DLPFC and the right SFG compared to the baseline. From the perspective of metabolite changes, this study further verified that DLPEC is affected by TLE and alteration in metabolism and brain network, but it cannot accurately locate the epileptogenic side like metabolite alteration in MTLC.

There were no significant associations for any metabolite with ages, age at epilepsy onset, duration of epilepsy, or interval days since the most recent seizure. Similarly, Simister et al.’s study (Simister et al., 2003), which used Proton MRS reveals frontal lobe metabolite abnormalities in idiopathic generalized epilepsy, there were no significant associations for any metabolite or metabolite ratio including NAA, Cho, Glx, Cr, and Ins against duration of epilepsy, or interval since the most recent TCS. Although Specifically, GABA levels can vary rapidly within 24 h after the expression of seizures or even after ingesting antiepileptic drugs (Kuzniecky et al., 2002). But the balance of excitation and inhibition would allow GABA to return to relatively stable levels without large fluctuations over more than 24 h. Studies on seizure patterns *in vivo* and *in vitro* have shown that excitatory neurons and inhibitory neurons try to suppress seizures by regulating their own activity, which may be a protective mechanism of the body itself (Schevon et al., 2012).

The lateralization of the epileptic side in patients with TLE, particularly in cases without remarkable findings on high-resolution MRI, remains challenging. Eventually, our findings revealed a trend and rule for metabolite changes in epileptic foci in patients with TLE, which can be verified and elaborated through ^1^H-MRS. Nonetheless, the association between epileptic discharges and GABA metabolic abnormalities requires further investigation, particularly regarding GABA distribution and concentration changes in the epileptic foci and other brain areas in different stages of epilepsy.

Our study demonstrated that MEGA-PRESS can be applied as an important additional method for diagnosing focal epilepsy, which is MRI-negative. MTLC GABA, NAA, NAA + NAAG, and Glu level reduction can lateralize the epileptic focus consistent with interictal video-EEG lateralization. TCS frequency is related to changes in GABA levels. GABA is a potential imaging biomarker for lateralizing the epileptic foci and monitoring therapeutic outcomes.

## Data availability statement

The raw data supporting the conclusions of this article will be made available by the authors, without undue reservation.

## Ethics statement

The studies involving human participants were reviewed and approved by the Ethics Department of the Second Affiliated Hospital of Xiamen Medical College. The patients/participants provided their written informed consent to participate in this study.

## Author contributions

GY, RW, and SW: study concept and design. SW, QW, HZ, and YZ: acquisition of data. SW: drafting of the manuscript. SW, QW, and DX: statistical analysis. GY and RW: study supervision. All authors contributed to the article and approved the submitted version.

## Funding

This study has received funding from the National Natural Science Foundation of China (Grant no. 31870981).

## Conflict of interest

The authors declare that the research was conducted in the absence of any commercial or financial relationships that could be construed as a potential conflict of interest.

## Publisher’s note

All claims expressed in this article are solely those of the authors and do not necessarily represent those of their affiliated organizations, or those of the publisher, the editors and the reviewers. Any product that may be evaluated in this article, or claim that may be made by its manufacturer, is not guaranteed or endorsed by the publisher.

## Glossary

^1^H-MRS: Proton magnetic resonance spectroscopy
AED: Anti-epileptic drug
DLPFC: Dorsolateral prefrontal cortex
EEG: Electroencephalography
FLAIR: Fluid-attenuated inversion recovery
FOV: Field of view
FSE: Fast spin-echo
GABA: γ-Aminobutyric acid
Gln: Glutamine
Glu: Glutamate
HCs: Healthy controls
IEEG: Intracranial electroencephalography
MEGA-PRESS: Mescher–Garwood point resolved spectroscopy sequence
MRI: Magnetic resonance imaging
MTLC: Mesial temporal cortex
NAA: N-acetylaspartate
NAAG: N-Acetylaspartylglutamate
NEX: Number of excitations
SD: Standard deviation
TCS: Tonic–clonic seizure
TE: Echo time
TLE: Temporal lobe epilepsy
TR: Repetition time
