# Supplementary material for: γ-Aminobutyric acid as a biomarker of the lateralizing and monitoring drug effect in patients with magnetic resonance imaging-negative temporal lobe epilepsy
Source: Front Neurosci. 2023 May 15;17:1184440. doi: 10.3389/fnins.2023.1184440 (PMC10225511; doi:10.3389/fnins.2023.1184440)

**Supplementary Material**

**Table S1 Clinical details of patients**

| Patients nos. | Gender | Age | Age of onset (years) | Duration of epilepsy  (years) | TCS  frequency  Per month | Interval time since the most recent seizure (months) | AED medications (daily dose g) | Interictal Video-EEG  Lateralization |
| --- | --- | --- | --- | --- | --- | --- | --- | --- |
| 1 | F | 31 | 20 | 11 | 16 | 0.25 | FYC 2 | L temp |
| 2 | M | 39 | 39 | 1 | 0.0625 | 0.16 | LEV 1 | R temp |
| 3 | M | 18 | 13 | 5 | 0 | 60 | LEV 1 | L>R temp |
| 4 | F | 20 | 17 | 3 | 0.33 | 0.07 | Irregular medication | L temp |
| 5 | M | 18 | 15 | 3 | 0.25 | 12 | LEV 1 + OXC 0.6 | R temp |
| 6 | F | 55 | 54 | 1 | 0.5 | 1 | Irregular medication | R temp |
| 7 | F | 22 | 20 | 2 | 0.5 | 3 | OXC 1.2 + LEV 2 | R>L temp |
| 8 | M | 21 | 20 | 1 | 0.5 | 0.25 | LEV 1 | R temp |
| 9 | F | 19 | 12 | 7 | 2 | 1 | Irregular medication | R>L temp |
| 10 | F | 52 | 40 | 12 | 3 | 0.03 | Irregular medication | R temp |
| 11 | M | 33 | 33 | 1 | 1 | 0.03 | LEV 1.5 | L temp |
| 12 | M | 22 | 19 | 3 | 0.33 | 12 | LEV 0.5 + VPA 0.5 | R temp |
| 13 | F | 23 | 13 | 10 | 2 | 1 | OXC 0.6 | R temp |
| 14 | M | 26 | 23 | 3 | 0.083 | 12 | OXC 1.2 + LEV 1 | R>L temp |
| 15 | M | 33 | 29 | 4 | 0.083 | 12 | OXC 1.2 | R temp |
| 16 | F | 30 | 27 | 3 | 0.2 | 2 | OXC 1.2 | L>R temp |
| 17 | F | 39 | 17 | 22 | 0.5 | 1 | VPA + LMT 0.0125  Irregular medication | R temp |
| 18 | M | 30 | 28 | 2 | 2 | 1 | LEV1.5 + FYC 6 | R temp |
| 19 | M | 53 | 14 | 39 | 0.83 | 1 | OXC 0.9 + FYC 0.004 | L temp |
| 20 | M | 34 | 28 | 6 | 0.083 | 12 | LMT 0.1 | L>R temp |
| 21 | M | 24 | 24 | 1 | 0.167 | 2 | OXC 0.3 | R>L temp |
| 22 | M | 25 | 21 | 4 | 0.083 | 1 | VPA 1 | R temp |
| 23 | F | 27 | 26 | 1 | 0.4 | 0.33 | LEV 1 | R>L temp |
| 24 | F | 24 | 20 | 4 | 0.17 | 6 | LEV 1 +FYC 0.006 + LMT 0.075 | R>L temp |
| 25 | M | 54 | 13 | 41 | 0.33 | 2.33 | LMT 0.075 + FYC 0.008 + VPA 1 | L temp |
| 26 | M | 26 | 18 | 8 | 0 | 60 | VPA 1 | R>L temp |
| 27 | M | 34 | 28 | 8 | 0.083 | 12 | LMT 0.1  Irregular medication | L>R temp |
| 28 | M | 24 | 24 | 1 | 0.5 | 2 | OXC 0.3+ LMT 0.1 +VPA 0.5  Irregular medication | R>L temp |
| 29 | M | 30 | 28 | 2 | 1 | 2 | FYC 0.008 + LEV 1.5 | R>L temp |
| 30 | M | 31 | 28 | 3 | 2 | 1 | FYC 0.008 + LEV 1.5 | R>L temp |
| 31 | F | 22 | 17 | 5 | 0.083 | 12 | LMT 0.075 | L temp |
| 32 | M | 23 | 23 | 1 | 1 | 0.33 | None | R>L temp |
| 33 | F | 22 | 20 | 3 | 0.083 | 0.03 | None | R>L temp |
| 34 | M | 29 | 26 | 3 | 2 | 0.13 | VPA- Interruption | R temp |
| 35 | M | 48 | 42 | 6 | 2 | 0.33 | VPA | R temp |
| 36 | F | 32 | 28 | 4 | 0 | 36 | CBZ | L temp |
| 37 | M | 34 | 32 | 2 | 0.083 | 4 | None | R temp |

R, right; L, left; CBZ, carbamazepine; FYC, Fycompa; LEV, Levetiracetam; OXC, Oxcarbazepine; LMT, Lamotrigine; VPA, Sodium valproate; EEG, electroencephalography; AED, antiepileptic drug; temp, temporal; TCS, tonic-clonic seizure; AED, Antiepileptic Drug; None, not taking AED

**Table S2-S4**

**Linear regression analysis of clinical indicators (gender, age of patients, age of initial seizure, duration of disease, TCS frequency, the interval days since the last TCS) and GABA levels in epileptic MTLC.**


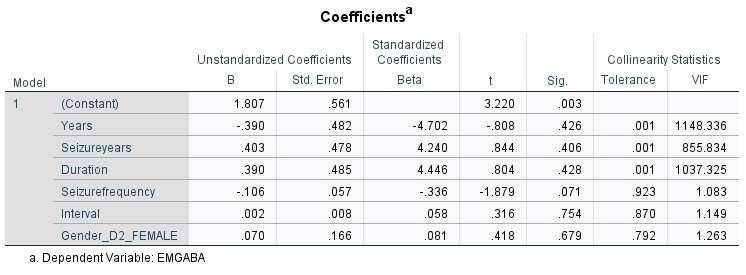


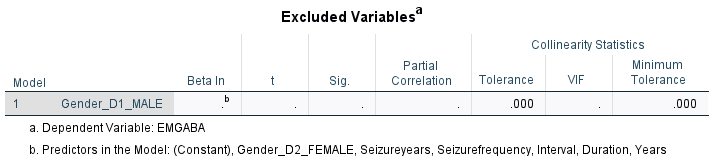


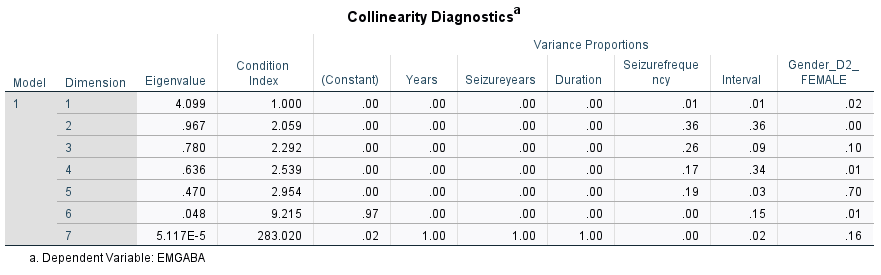

Supplement: Supplementary file 1 [file Data_Sheet_1.docx]
